# Reliability of the Strengths and Difficulties Questionnaire in Japanese Preschool Children Aged 4–6 Years

**DOI:** 10.2188/jea.JE20140050

**Published:** 2014-11-05

**Authors:** Yuriko Doi, Kaneyoshi Ishihara, Makoto Uchiyama

**Affiliations:** 1 Area on Epidemiological Research, National Institute of Public Health, Wako, Japan; 1 国立保健医療科学院疫学調査研究分野; 2 Department of Child Welfare, Notre Dame Seishin University, Okayama, Japan; 2 ノートルダム清心女子大学人間生活学部児童学科; 3 Department of Psychiatry, Nihon University School of Medicine, Tokyo, Japan; 3 日本大学医学部精神医学系

**Keywords:** strengths and difficulties questionnaire, SDQ, reliability, preschool children, Japan

## Abstract

**Background:**

The Strengths and Difficulties Questionnaire (SDQ) has been widely used as a brief behavioral screening. The aim of this study was to examine the internal consistency and test-retest reliability of the 3- to 4-year-old version of the SDQ (SDQ 3–4) in Japanese preschool children.

**Methods:**

The SDQ 3–4 was administered to 754 parents who had 4- to 6-year-old children attending kindergartens or childcare centers in Wako City, Japan, at 2 different times (Time 1 and Time 2) over a 2-week interval between June and July 2012. Cronbach’s α and correlation coefficients were used to examine internal consistency and test-retest reliability, respectively.

**Results:**

Of 393 parents who returned their responses at Time 1 (response rate 52.1%), 383 were used for analysis after excluding 10 responses with missing data. Their children’s mean age was 4.7 (standard deviation 0.7) years. The internal consistency (Cronbach’s α) was good for the total difficulties score (0.74) and the prosocial behavior scale (0.70). However, it was slightly worse for the emotional symptoms, conduct problems, and hyperactivity scales (0.61–0.66) and poor for the peer problems scale (0.45). Of the 383 included respondents at Time 1, 211 parents returned their responses at Time 2 (response rate: 55.1%). Test-retest reliability (correlation coefficients) was good (0.73–0.82), except for the peer problems scale (0.58).

**Conclusions:**

The results support the reliability of the SDQ 3–4 being satisfactory for the total difficulties score and prosocial behavior scale and being acceptable for the emotional symptoms, conduct problems, and hyperactivity scales in Japanese preschool children aged 4–6 years.

## Abstract (ja)

**背景：:**

Strengths and Difficulties Questionnaire（SDQ）は、広く用いられている簡便な行動スクリーニングテストである。本研究の目的は、日本の就学前の子どもにおける3～4歳児用SDQ（SDQ 3—4）の信頼性（内部的一貫性およびテスト‐再テスト）について検討を行うことであった。

**方法：:**

和光市の幼稚園または保育所に通う就学前の4歳から6歳の子ども754人の保護者に対し、2012年6月から7月にかけて2度（Time 1とTime 2）、SDQ 3—4を用いた調査を実施した。内部的一貫性についてはクローンバックのα係数、テスト‐再テストについては両テスト間の相関係数を用いて検討した。

**結果：:**

Time 1で回答した393人の保護者（回答率52.1%）のうち解析に用いた383人について、クローンバックのα係数は、Total Difficultiesスコアで0.74、向社会性サブスケールスコアで0.70であった。しかし、情緒、行為、多動・不注意のサブスケールススコアは0.61～0.66、仲間関係では0.45であった。383人のうち、211人の保護者はTime 1とTime 2の両方に回答した（55.1%）。Time 1とTime 2の両テスト間の相関係数は、仲間関係（0.58）を除き、Total Difficultiesスコアおよび他のサブスケールスコアでは0.73～0.82であった。

**結論：:**

以上より、日本の就学前の4歳から6歳の子どもにおいて、SDQ 3—4は、Total Difficultiesスコアおよび向社会性、情緒、行為、多動・不注意の各サブスケールススコアで、信頼性のある尺度であることが示唆された。

## INTRODUCTION

The Strengths and Difficulties Questionnaire (SDQ) is a brief behavioral screening questionnaire that provides balanced coverage of behaviors, emotions, and relationships for children and adolescents aged 4–16 years.^1^ Since its development, the SDQ has been translated into many languages,^2^ extensively examined for its psychometric properties,^3^ and widely used as a screening and research tool in clinical and community populations.^3^ However, few studies have investigated its psychometric properties for preschool-aged children.^3^^–^^6^

In psychometric theory, the concept of reliability is a fundamental way to reflect the amount of measurement error. It is important to identify and quantify multiple potential sources of error that cause performance to vary across items within a test (internal consistency) and among different observers (inter-observer reliability), and that appear when different forms of a test are used at different times (test-retest reliability).^7^^,^^8^

The SDQ has two age versions: the SDQ 3–4 and the SDQ 4–16. We adopted the SDQ 3–4 for young children around the age of 4 years in our study, based on our experience and literature review. The aim of this study was to examine the internal consistency and test-retest reliability of the SDQ 3–4 in Japanese preschool children aged 4–6 years on the basis of their parents’ reports.

## METHODS

### Subjects and data collection

We conducted a survey of Children’s Sleep and Health between June and July 2012.^9^ Study subjects included children aged 4–6 years who had entered any of 4 kindergartens or 11 childcare centers at the age of 4 or 5 years as of April 2, 2012, in Wako City in the Tokyo Metropolitan Area. We sent a letter to the principal of each kindergarten and childcare center requesting their participation and indicating the purpose and procedure of our study. Eventually, three kindergartens and eight childcare centers agreed to participate.

The teachers at these institutions were provided legal-sized envelopes that contained an invitation letter, two forms of parent-report anonymous questionnaires with anonymous study numbers (one in yellow and the other in blue), two self-addressed stamped envelopes, and a ballpoint pen. These items were then provided to all the children’s parents by the teachers. Of 754 parents, 393 agreed to participate in the study.

The yellow form comprised five sections: (a) sociodemographic information; (b) children’s sleep patterns, including the Children’s ChronoType Questionnaire (CCTQ)^10^^,^^11^; (c) children’s daytime activities; (d) children’s behavior assessed using the SDQ^1^^,^^12^^,^^13^; and (e) parents’ diurnal preferences using the Morningness/Eveningness (M/E) scale^14^ modified for parents. The blue form comprised the CCTQ, the SDQ, and the M/E scale for parents, to examine the temporal stability of these measures.

For the first survey (Time 1), one of the child’s parents or guardians was instructed to fill out the yellow form and immediately mail it in the stamped and self-addressed envelope to the principal investigator. For the second survey (Time 2), they were requested to do the same thing, using the blue form at an interval of two weeks.

The Institutional Review Board of the National Institute of Public Health, Japan, approved this study, which was conducted in accordance with the principles articulated in the Declaration of Helsinki of 1975.

### SDQ

The SDQ includes 25 items divided into 5 scales of 5 items each. The scales assess emotional symptoms, conduct problems, hyperactivity, peer problems, and prosocial behavior. The score for each scale is generated by summing the scores for the five items comprising that scale, thereby generating a scale score ranging from 0 to 10. The scores for the emotional symptoms, conduct problems, hyperactivity, and peer problems scales can be summed to generate a total difficulties score ranging from 0 to 40; the prosocial behavior scale score is not incorporated into the total difficulties score since the absence of prosocial behaviors is conceptually different from the presence of psychological difficulties. Higher scores represent more emotional and behavioral problems for the emotional symptoms, conduct problems, hyperactivity, and peer problems scales, as well as for the total difficulties score; on the other hand, higher scores represent more positive prosocial behaviors for the prosocial behavior scale.^1^^,^^12^

There are two age versions of the SDQ: the SDQ 3–4 and the SDQ 4–16.^12^^,^^13^ The SDQ 3–4 is the same as the SDQ 4–16, except for Items 18 and 22 in the conduct problems scale and Item 21 in the hyperactivity scale. The respective items of 18 and 22 in the SDQ 4–16, referring to “often lies or cheats” and “steals from home, school, or elsewhere” are replaced by items referring to “often argumentative with adults” and “can be spiteful to others” in the SDQ 3–4. Item 21 is softened from “thinks things out before acting” in the SDQ 4–16 to “can stop and think things out before acting” in the SDQ 3–4.

As mentioned above, the majority of children in the present study were 4 years old or had just turned 5 years old. From the viewpoint of our experience in childcare and early childhood education, we regarded the phrasing of the items in the SDQ 3–4 as better suited for the children in our study than those in the SDQ 4–16. Furthermore, we reviewed the relevant literature on the SDQ and found that, although little research exists for children around the age of 4 years, the Longitudinal Study of Australian Children used the SDQ 3–4 for 4–5-year-old children.^15^ Therefore, we decided to use the SDQ 3–4 in our study.

### Statistical analysis

Cronbach’s α coefficient was calculated to assess the internal consistency of the scale for participants at Time 1. A value >0.70 was deemed satisfactory, while values between 0.60 and 0.70 are generally considered to be acceptable. To further assess homogeneity of the scale, the corrected item-total correlation was used. A value >0.2 was assumed to be acceptable for homogeneity of the scale.

The subjects who participated in this study at Times 1 and 2 were aggregated using anonymous study numbers indicated on the covers of the questionnaire forms. Pearson’s correlation coefficient or Spearman’s ρ correlation coefficient was used, depending on whether the scores were normally distributed, to examine temporal stability (test-retest reliability). A *P* value <0.05, calculated using a two-sided test, was considered statistically significant.^7^^,^^8^ IBM SPSS Statistics 20 (SPSS Japan, Tokyo, Japan) was used to perform all statistical analyses.

## RESULTS

Of 754 subjects, 393 returned their responses at Time 1 (response rate, 52.1%). After excluding 10 respondents with missing values for children’s sex or age, responses from 383 were used as participants at Time 1: 378 mothers, 4 fathers, and 1 grandmother. Of the 383 participants at Time 1, 211 returned their responses at Time 2 (response rate, 55.1%). Table 1 shows the characteristics of participants who responded at Time 1 as well as those who responded at Times 1 and 2, which were almost the same. Children’s mean age was 4.7 (standard deviation [SD] 0.7) years, and over 85% of the heads of family were office workers or public officers.

**Table 1.** Characteristics of preschool children and their families

Characteristics	Participants at Time 1		Participants at Times 1 and 2	
	(*n* = 383)		(*n* = 211)	
	
	*n*	%	*n*	%
Child’s sex				
Boy	190	49.6	97	46.0
Girl	193	50.4	114	54.0
Child’s age				
Mean (SD)	4.7 (0.7)		4.7 (0.7)	
4 years	153	39.9	87	41.2
5 years	182	47.5	98	46.4
6 years	48	12.5	26	12.3
Birth order				
1st	202	52.7	121	57.3
2nd	141	36.8	71	33.6
3rd, 4th, or 5th	39	10.2	16	7.6
Unknown	1	0.3	3	1.4
Attending				
Kindergarten	223	58.2	90	42.7
Childcare center	160	41.8	121	57.3
Parent’s age, Mean (SD)				
Father	38.4 (5.3)		38.5 (4.8)	
Mother	36.6 (4.7)		37.0 (4.6)	
Parent’s occupation				
Office worker	255	66.6	147	69.7
Public officer	68	17.8	33	15.6
Worker in other services	6	1.6	2	0.9
Self-employed worker	33	8.6	15	7.1
Others	21	5.6	14	6.6
Family structure				
Two-generation family	349	91.1	192	91
Three-generation family	18	4.7	11	5.2
Unknown	16	4.2	8	3.8

SD, standard deviation.

To examine test-retest reliability, the parent-report questionnaires were administered at the two different times, Time 1 and Time 2, over approximately 2 weeks (Mean 16.4 [SD 6.7] days).

Table 2 shows the internal consistency of the SDQ subscales. Cronbach’s α for the total difficulties score and the prosocial behavior scale exceeded 0.7. Cronbach’s α for the hyperactivity, conduct problems, and emotional symptoms scales lay between 0.7 and 0.6, whereas Cronbach’s α for the peer problems scale was 0.45. Cronbach’s α for the hyperactivity scale improved when Item 10 was deleted. Corrected item-total correlation coefficients for Item 10 in the hyperactivity scale, and for Items 11 and 19 in the peer problems scale were <0.20. The remaining corrected item-total correlation coefficients were >0.20. Similar results were found for boys and girls, although Cronbach’s α for the peer problems and emotional symptoms scales were slightly lower in girls than in boys. More items with item-total correlation coefficients of <0.2 in the peer problems scale were identified in girls than in boys.

**Table 2.** Homogeneity and internal consistency of the 3- to 4-year-old verion of Strengths and Difficulties Questionnaire (SDQ 3–4) for preschool children at Time 1 (*n* = 383)

SDQ scale	*n*	Both		*n*	Boys		*n*	Girls	
		
		Corrected item-total correlation	α^d^		Corrected item-total correlation	α^d^		Corrected item-total correlation	α^d^
Emotional symptoms^a^									
Q3	380	0.26	0.60	193	0.30	0.61	187	0.23	0.60
Q8		0.33	0.58		0.38	0.58		0.29	0.57
Q13		0.34	0.57		0.36	0.58		0.33	0.55
Q16		0.44	0.52		0.45	0.54		0.42	0.50
Q24		0.48	0.49		0.48	0.52		0.50	0.44
Overall			**0.61**			**0.63**			**0.59**
Conduct problems^a^									
Q5	381	0.49	0.50	192	0.48	0.52	189	0.50	0.49
Q7		0.30	0.60		0.30	0.61		0.33	0.59
Q12		0.34	0.58		0.40	0.57		0.27	0.61
Q18		0.43	0.54		0.35	0.59		0.52	0.47
Q22		0.35	0.58		0.43	0.56		0.27	0.61
Overall			**0.62**			**0.63**			**0.62**
Hyperactivity^a^									
Q2	379	0.49	0.57	189	0.52	0.56	190	0.46	0.58
Q10		0.12	0.70		0.19	0.69		0.06	0.72
Q15		0.57	0.52		0.65	0.48		0.52	0.55
Q21		0.43	0.60		0.36	0.63		0.48	0.57
Q25		0.44	0.59		0.36	0.63		0.52	0.55
Overall			**0.66**			**0.66**			**0.66**
Peer problems^a^									
Q6	383	0.25	0.39	193	0.30	0.43	190	0.19	0.35
Q11		0.17	0.44		0.16	0.51		0.17	0.36
Q14		0.23	0.41		0.25	0.46		0.20	0.34
Q19		0.19	0.43		0.22	0.48		0.16	0.37
Q23		0.37	0.30		0.46	0.32		0.27	0.27
Overall			**0.45**			**0.50**			**0.39**
Total difficulties^a,b^									
Overall	374		**0.74**	188		**0.76**	186		**0.73**

Prosocial behavior^c^									
Q1	383	0.48	0.64	193	0.48	0.62	190	0.46	0.65
Q4		0.43	0.66		0.38	0.66		0.49	0.64
Q9		0.56	0.60		0.54	0.59		0.58	0.60
Q17		0.40	0.67		0.43	0.64		0.37	0.69
Q20		0.40	0.67		0.39	0.66		0.40	0.68
Overall			**0.70**			**0.69**			**0.70**

The parent-report questionnaires including the SDQ 3–4 were administered at Time 1.

^a^Higher scores (range: 0–10) represent more emotional and behavioral problems.

^b^The total difficulties score (range: 0–40) indicates the sum of the emotional symptoms, conduct problems, hyperactivity, and peer problems scales.

^c^Higher scores (range: 0–10) represent more prosocial behaviors.

^d^Cronbach’s α coefficients are represented for all items included as well as each item deleted. Overall Cronbach’s α coefficients are indicated by boldface.

Table 3 shows the test-retest reliability of the SDQ. The mean time interval between the first and second observations was 16.4 (SD 6.7) days. The scores at the two different times (Time 1 and Time 2) were highly correlated for all SDQ scales as well as the total difficulties score. However, the correlation coefficient for the peer problems scale was slightly lower than for the other scales, although still statistically significant.

**Table 3.** Test-retest reliability of the 3- to 4-year-old verion of Strengths and Difficulties Questionnaire (SDQ 3–4) for preschool children at Times 1 and 2 (*n* = 211)

SDQ scale	Both		Boys		Girls	
		
	*n*	Correlation coefficients^d^	*n*	Correlation coefficients^d^	*n*	Correlation coefficients^d^
Emotional symptoms^a^	208	0.77*	97	0.83*	111	0.73*
Conduct problems^a^	208	0.73*	96	0.74*	112	0.73*
Hyperactivity^a^	208	0.77**	94	0.84**	114	0.70**
Peer problems^a^	211	0.58*	97	0.57*	114	0.58*
Total difficulties^a,b^	202	0.82**	93	0.82**	109	0.81**

Prosocial behavior^c^	208	0.76**	96	0.81**	112	0.73**

^a^Higher scores represent more emotional and behavioral problems.

^b^The total difficulties indicates the sum of the emotional symptoms, conduct problems, hyperactivity, and peer problems scales.

^c^Higher scores represent more prosocial behaviors.

^d^Correlations between the scores at the first (Time 1) and second (Time 2) observations. The mean time interval between the observations was 16.4 (standard deviation 6.7) days.

*P < 0.05. **P < 0.01.

## DISCUSSION

To the best of our knowledge, the present study is the first to examine the reliability of the SDQ 3–4 in Japanese preschool children. The results show that the SDQ 3–4 is generally reliable, but some items and scales require careful attention during use and interpretation.

As described above in the methods, Items 18 and 22 in the conduct problems scale of the SDQ 3–4 were different in wordings from those in the SDQ 4–16. The corrected item-total correlations for Items 18 and 22 were both >0.2. After these items were excluded from this scale, the respective Cronbach’s α (0.54 and 0.58) was lower than the overall Cronbach’s α (0.62). Taken together, these findings suggest that the conduct problems scale is homogenous and internally consistent. Cronbach’s α for the conduct problems scale in the present study was comparable to the value of 0.64 in Dutch children aged 3–4 years obtained in a previous study using the SDQ 3–4.^6^ In addition, its test-retest reliability was good.

Item 21 in the hyperactivity scale of the SDQ 3–4 was slightly different in wording from that in the SDQ 4–16. The corrected item-total correlation of Item 21 was >0.2. After this item was excluded from the hyperactivity scale, Cronbach’s α (0.60) was lower than the overall Cronbach’s α (0.66). On the other hand, Item 10, which relates to constantly fidgeting or squirming, did not significantly correlate with the scale total omitting that score, and the Cronbach’s α increased from 0.66 to 0.70 when this item was deleted. These findings suggest that Item 10 may differ from the remaining items, potentially tapping different aspects of the same attribute. Further research is required to examine if these findings are reproducible; if so, how responses to this item differ between Japan and other countries from the viewpoint of cross-cultural psychology warrants further study.

The Cronbach’s α of the hyperactivity scale in the present study (0.66) was slightly lower than those reported in Dutch children aged 3–4 years (0.74)^6^ and 5–6 years (0.78),^4^ as well as Finnish children aged 4–6 years (0.77).^5^ For Japanese children aged 4–15 years, Cronbach’s α was 0.75–0.76, but preschool children aged 4–6 years represented only 3% of children included in the sample.^16^^,^^17^ Overall, Cronbach’s α for this scale in the present study was at an acceptable level, and its test-retest reliability was good.

For items in the peer problems scale, the internal consistency (Cronbach’s α) was poor in the present study (0.39–0.50). This finding is comparable with those (0.42–0.57) in Dutch and Finish children aged 3–6 years^4^^–^^6^ as well as that (0.53) in children aged 4–12 obtained from 26 studies,^3^ including one of Japanese children.^17^ Each item was not highly correlated with the scale total omitting that score, especially Item 11 (having at least one good friend) and Item 19 (being picked on or bullied by other children). Furthermore, the test-retest reliability of this scale was not as good as that of the other scales. These peer relationships among children may be difficult for parents to observe and identify. As pointed out in previous studies,^3^^,^^5^ peer problems are most often assessed on the basis of reports by children themselves, which may not be known to parents. They may sometimes be reported by teachers at kindergartens or childcare centers. Therefore, it seems that the items in the peer problems scale may be difficult for parents to assess in a reliable or stable way not only at the present time but also a later time.

A few limitations to the present study should be taken into account. First, the subjects studied may not be representative of preschool children in Japan because the present study was conducted in a geographically limited area. Second, the response rate was 52%, which implies that there may be non-response bias to some extent. Last, the inter-observer agreement between parent and teacher ratings (inter-observer reliability) was not examined because of practical difficulties.

In conclusion, the results of this study support the notion that the internal consistency and test-retest reliability of the SDQ 3–4 is satisfactory for the total difficulties score and the prosocial behavior scale and is acceptable for the emotional symptoms, conduct problems, and hyperactivity scales in Japanese preschool children aged 4–6 years.

## ONLINE ONLY MATERIAL

Abstract in Japanese.
