# Supplementary material for: Reliability of the Strengths and Difficulties Questionnaire in Japanese Preschool Children Aged 4–6 Years
Source: J Epidemiol. 2014 Nov 5;24(6):514–8. doi: 10.2188/jea.JE20140050 (PMC4213227; doi:10.2188/jea.JE20140050)
Supplement: Abstract in Japanese. [file je-24-514-s001.pdf]

日本の就学前の 4～6 歳の子どもにおける Strengths and Difficulties Questionnaire の信頼性

土井由利子[1]、石原金由[2]、内山真[3]
[1] 国立保健医療科学院疫学調査研究分野、[2] ノートルダム清心女子大学人間生活学部児童学科、[3] 日本大学医学部精神医学系

背景：Strengths and Difficulties Questionnaire（SDQ）は、広く用いられている簡便な行動スクリーニングテストである。本研究の目的は、日本の就学前の子どもにおける 3～4 歳児用 SDQ（SDQ 3−4）の信頼性（内部的一貫性およびテスト‐再テスト）について検討を行うことであった。
方法：和光市の幼稚園または保育所に通う就学前の 4 歳から 6 歳の子ども 754 人の保護者に対し、2012 年 6 月から 7 月にかけて 2 度（Time 1 と Time 2）、SDQ 3−4 を用いた調査を実施した。内部的一貫性についてはクローンバックの α 係数、テスト‐再テストについては両テスト間の相関係数を用いて検討した。
結果：Time 1 で回答した 393 人の保護者（回答率 52.1%）のうち解析に用いた 383 人について、クローンバックの α 係数は、Total Difficulties スコアで 0.74、向社会性サブスケールスコアで 0.70 であった。しかし、情緒、行為、多動・不注意のサブスケールススコアは 0.61～0.66、仲間関係では 0.45 であった。383 人のうち、211 人の保護者は Time 1 と Time 2 の両方に回答した（55.1%）。Time 1 と Time 2 の両テスト間の相関係数は、仲間関係（0.58）を除き、Total Difficulties スコアおよび他のサブスケールスコアでは 0.73～0.82 であった。
結論：以上より、日本の就学前の 4 歳から 6 歳の子どもにおいて、SDQ 3−4 は、Total Difficulties スコアおよび向社会性、情緒、行為、多動・不注意の各サブスケールススコアで、信頼性のある尺度であることが示唆された。

キーワード
Strengths and Difficulties Questionnaire、SDQ、信頼性、就学前のこども（就学前児童）、日本
